# Cancer-Associated Fibroblasts Promote the Upregulation of PD-L1 Expression Through Akt Phosphorylation in Colorectal Cancer

**DOI:** 10.3389/fonc.2021.748465

**Published:** 2021-11-19

**Authors:** Yang Gao, Zhao Sun, Junjie Gu, Zhe Li, Xiuxiu Xu, Chunling Xue, Xuechun Li, Lin Zhao, Jianfeng Zhou, Chunmei Bai, Qin Han, Robert Chunhua Zhao

**Affiliations:** ^1^ Department of Oncology, Peking Union Medical College Hospital, Chinese Academy of Medical Sciences and Peking Union Medical College, Beijing, China; ^2^ Department of Gynecologic Oncology, National Cancer Center/National Clinical Research Center for Cancer/Cancer Hospital, Chinese Academy of Medical Sciences and Peking Union Medical College, Beijing, China; ^3^ Institute of Basic Medical Sciences Chinese Academy of Medical Sciences, School of Basic Medicine Peking Union Medical College, Peking Union Medical College, Beijing, China; ^4^ School of Life Sciences, Shanghai University, Shanghai, China

**Keywords:** colorectal cancer, cancer-associated fibroblasts, PD-L1, Akt phosphorylation, immune escape

## Abstract

Upregulation of immune checkpoint proteins is one of the main mechanisms for tumor immune escape. The expression of programmed death ligand-1 (PD-L1) in colorectal cancer (CRC) is higher than in normal colorectal epithelial tissue, and patients with higher PD-L1 expression have a poorer prognosis. Additionally, PD-L1 expression in CRC is affected by the tumor microenvironment (TME). As a major component of the TME, cancer-associated fibroblasts (CAFs) can act as immune regulators and generate an immunosuppressive tumor microenvironment. Therefore, we speculated that CAFs may be related to the upregulation of PD-L1 in CRC, which leads to tumor immune escape. We found that CAFs upregulate PD-L1 expression in CRC cells through AKT phosphorylation, thereby reducing the killing of CRC cells by peripheral blood mononuclear cells. The ratio of CAFs to CRC cells was positively correlated with AKT phosphorylation and the expression of PD-L1 in CRC *in vitro.* Consistent with the *in vitro* results, high CAF content and high expression of PD-L1 were negatively correlated with disease-free survival (DFS) of CRC patients. These results indicate that the upregulation of PD-L1 expression in CRC by CAFs through the activation of Akt is one of the molecular mechanisms of tumor immune escape. Thus, targeted anti-CAF therapy may help improve the efficacy of immunotherapy.

## Introduction

Colorectal cancer (CRC) is one of the most common malignancies around the world, ranking third in overall incidence and fourth in terms of cancer-related mortality (1, 2). In spite of continuous improvements in CRC therapy, the average 5-year survival of cancer patients at all stages is 45-60% (2, 3). Recent studies have found that the tumor immune microenvironment, infiltration of immune cells, inflammatory cytokines, and expression of immune checkpoint proteins greatly impact the survival of CRC patients (4–9).

As one of the most important immune checkpoint proteins, programmed death ligand-1 (PD-L1) is upregulated in CRC and its high expression is corelated with a poor prognosis, suggesting that PD-L1 may be involved in the progression of CRC (4, 8, 10). The binding of PD-L1 to PD-1 inhibits T cell function and promotes tumor evasion (11). The upregulation of PD-L1 is one of the most important and most widely studied mechanisms of immune escape (12). Blocking the PD-1/PD-L1 axis can improve the efficacy of immunotherapy, and improve the survival of patients (4, 13–15). Therefore, it is warranted to study the mechanism of PD-L1 upregulation in CRC, which may provide new therapeutic strategies.

Upregulation of PD-L1 expression in tumor cells may be affected by the tumor microenvironment (TME) (16–18). As one of its most abundant components, cancer-associated fibroblasts (CAFs) can affect the TME by secreting a variety of chemokines, cytokines, and growth factors, as well as regulating the composition of the extracellular matrix, leading to immunosuppression and tumor progression (19, 20). Previous studies have shown that CAFs can promote the growth and survival of tumor cells as well as tumor angiogenesis in CRC, leading to a poor prognosis (21–23). CAFs are proven to upregulate PD-L1 expression in tumor cells, but the underlying mechanism remains insufficiently clear (16–18). In our previous study, we also found that the expression of α-smooth muscle actin [α-SMA, a marker gene of CAFs (24)] is positively correlated with PD-L1 expression in clinical CRC samples. Consequently, we further explored the mechanism by which CAFs regulate the expression of PD -L1, thus elucidating a possible mechanism through which CAFs promote the immune escape of CRC.

## Materials and Methods

### Isolation and Culture of Human Adipose Tissue Derived Mesenchymal Stem Cells (hAD-MSCs)

Human adipose tissue was collected from the plastic surgery department of Peking Union Medical College Hospital (PUMCH, Beijing, China) with the donors’ informed consent. Then, hAD-MSCs were isolated and cultured according to the method described in our previous study (25). The adipose tissue was washed with D-Hanks’ buffer and centrifuged at 1,000 g for 3min. The adipose tissue below the liquid surface was transferred to a fresh centrifugal tube, washed, digested with 0.2% collagenase P (Life Technology Corporation, USA) and incubated at 37°C for 30min. The undigested adipose tissue was removed with a 100-μm cell strainer. Then, an appropriate amount of D-Hanks’ buffer was added and centrifuged at 1500g for 10min. The supernatant was discarded and the pellet was washed twice with phosphate buffered saline (PBS). Then, the cells were collected through centrifugation. Finally, 1 × 10^6^ cells were seeded into the culture medium and incubated at 37°C in a humidified atmosphere comprising 5% CO_2_. The culture medium was changed every 2-3 days. The cells were passaged or cryopreserved after reaching 80% confluence.

### Flow Cytometry

Flow cytometry was used to identify the immunophenotypes of hAD-MSCs according to a published method (25). Approximately 2 × 10^5^ hAD-MSCs were harvested, washed with PBS, and incubated with primary antibodies (CD31, CD34, CD106, CD29, CD44, CD73, CD90; BD Pharmingen, USA) at 4°C for 1h. After washing off the primary antibodies, the hAD-MSCs cells were incubated with a fluorescence-labeled secondary antibody (BD Pharmingen, USA) at 4°C for 30min. The immunopositive cells were quantified using an Accuri C6 Flow Cytometer (BD Biosciences, USA) and the data were analyzed using Cflow Plus Software (BD Biosciences, USA).

### Cultivation of CRC Cell Lines

Human CRC cell lines HCT116, HCT8 and LOVO were obtained from the Cell Resource Center, IBMS, CAMS/PUMC (Beijing, China). The cell lines were cultured in Dulbecco’s modified Eagle’s medium (DMEM) with high glucose (Gibco, USA) containing 10% fetal bovine serum (FBS) (Thermo Fisher Scientific, USA) at 37°C in a humidified atmosphere comprising 5% CO_2_.

### Extraction of Exosomes From CRC Cell Lines (CRC-Exosomes)

The HCT116/HCT8/LOVO cells were cultured in FBS-free DMEM/high glucose medium for 36-48 hours before exosome extraction. Then, the culture supernatants were collected and centrifuged at 3000g for 30min to remove cell debris and dead cells. The residual cell debris and large vesicles were removed by filtered through a 0.22-μm pore-size membrane. The filtered supernatant was sequential centrifuged at 120,000g for 2 hours at 4°C in an ultracentrifuge (Optima XPN-100, Beckman Coulter, USA). The sample was washed twice with moderate D-Hanks’ buffer. The remaining liquid was filtered through a 0.2-μm pore-size membrane, aliquoted into 1.5-ml sterile EP tubes and stored at -80°C (26).

### Transmission Electron Microscopy

The ultrastructure of exosomes was analyzed by transmission electron microscopy (TEM). The exosomes were collected and suspended in PBS. Then, the CRC-exosomes were fixed, dehydrated, embedded, sliced, stained with uranium acetate and lead citrate, and observed under a TEM as described previously (27).

### CRC-Exosome-Induced Differentiation of hAD-MSCs Into CAFs

When the hAD-MSCs adhered to the dish after cell passaging, the medium was replaced with FBS-free DMEM/F-12 medium (Gibco, USA) and changed every other day. CRC-exosomes were added to the medium to a final concentration of 50 mg/L on d0, d2, d4, d6 and d8, respectively. HAD-MSCs were confirmed to be induced to differentiate into CAFs on the 9th day.

### Adipogenic and Osteogenic Differentiation of hAD-MSCs and CAFs

When hAD-MSCs/CAFs grew to 90% confluence, the medium was replaced with adipogenic differentiation medium. The new culture medium was replaced at intervals of two days. Oil red O staining was performed on the 11th day of induction to detect the formation of lipid droplets in cells as described previously (25).

When hAD-MSCs/CAFs grew to 80% confluence, the spent medium was discarded, after which osteogenic differentiation medium was added and replaced at an interval of two days. Alizarin red staining was carried out on the 14th day of induction to detect extracellular mineralization. After incubating for 30 min at 37°C, the staining results were observed under a conventional optical microscope (25).

### Uptake of Exosomes by hAD-MSCs

CRC-exosomes were labeled with 1μM 1´-Dioctadecyl-3,3,3´,3-tetramethylindocarbocyanine perchlorate (Dil) (ThermoFisher, USA) and incubated for 10min. The labeled exosomes were centrifuged at 700,000 × g at 4°C for 40min. After discarding the supernatant, the exosomes were then added into hAD-MSCs and co-incubated in dark. The cells were washed 3 times with PBS after 4 hours, fixed in 4% paraformaldehyde for 10min, and stained with Hoechst 33342 (ThermoFisher, USA). After washing 3 times with PBS, the uptake of exosomes was observed under fluorescence microscope.

### Treatment of CRC Cells With CAF-CM

When the hAD-MSCs were induced to differentiate into CAFs after CRC-exosomes treatment on day9, the culture medium of CAFs was removed and the cells were rinsed with D-Hanks’ buffer. Then, the cells were cultured in DMEM/high glucose medium containing 10% FBS for 24 hours, and the resulting supernatant, also known as CAFs-conditioned medium (CAF-CM), was removed and aliquoted into 1.5ml EP tubes for cryopreservation at -80°C.

CRC cells (HCT116/HCT8/LOVO) were cultured in CAF-CM for 1h, 3h, 6h, 24h and 48h, respectively. Another group of CRC cells in high glucose medium was set as a control group. The cells were suspended in neutral Radio Immunoprecipitation Assay (RIPA) lysis buffer containing 1mM phenylmethanesulfonylfluoride with/without AKT phosphorylase inhibitor (pAKTi, MK-2206 2HCI, Celleck, USA) and collected into 1.5ml EP tubes. After centrifugation, the supernatant was collected and cryopreserved.

### *In Vitro* PBMCs Cytotoxicity Assay

Approximately 5×10^3^ CRC cells (HCT8, HCT116, LOVO) were seeded into 96-well plates and co-cultured with different proportions of peripheral blood mononuclear cells (PBMCs) from healthy donors for 3 days. Firstly, the target cells were added to the wells with culture medium and incubated for 4 hours so that the cells became adherent. Then, the effector cells were mixed in the wells according to the indicated effector: target (E:T) ratios. Wells containing only target cells were used as the positive control. The blank well was used as the background control. The cells were washed with PBS for 3 times, then, 20μl of MTS [3- (4, 5-dimethylthiazol-2-yl)-5- (3-carboxymethoxyphenyl)-2- (4-sulfophenyl)-2H-tetrazolium] (Promega, USA) was added to each plate and incubated at 37°C for 1h. The absorbance at 490nm (A_490_) was measured for each plate using a microplate reader (BioTek Epoch, USA). Each group was replicated in 6 wells. The survival of CRC cells was calculated based on the A_490_ value of each well. The cytotoxicity was calculated based on the survival of CRC cells. To further investigate the effect of Akt and PD-L1, pAKTi and PD-L1 blocker (αPD-L1) atezolizumab biosimilar (R&D System, USA) were used to pre-block pAkt and PD-L1 respectively. The cells were incubated with pAKTi/αPD-L1 for 72h and washed with PBS for 3 times before the cytotoxicity assay.


$$\%Cytotoxicicty=\left[1-\frac{A_{490}Experimental\;well-A_{490}Background}{A_{490}Positive\;control-A_{490}Background}\right]\times 100$$

### Analysis of Tumor Cell Apoptosis

Tumor cell apoptosis rate was detected according to the protocol of the Annexin V-FITC/PI Apoptosis Detection Kit (YEASEN, CHN). HCT116 cells were cultured with CAF-CM or normal medium for 48h. PBMCs were then added and co-cultured with tumor cells for 2 days. After HCT116 cells were treated with standard protocol, cell apoptosis rate was detected and analyzed using flow cytometry.

### Western Blot Analysis

Western blot analysis was done using the same protocol as in our previous study (28). Protein concentrations of cell lysates were determined using a BCA Protein Assay Kit (Beyotime, USA). Protein samples were separated by 10% acrylamide sodium dodecyl sulfate–polyacrylamide gel electrophoresis (SDS-PAGE) and transferred to polyvinylidene difluoride (PVDF) membranes (Millipore, USA). The membranes were blocked with skim milk in Tris-buffered saline with Tween 20 (TBST) for 1 hour and incubated overnight at 4°C with primary antibodies against PD-L1 (Abcam, UK), p-Akt (Cell Signaling Technology, USA), α-SMA (Cell Signaling Technology, USA), PD-1(Cell Signaling Technology, USA), and GAPDH (Cell Signaling Technology, USA). After washing with TBST, the membranes were incubated with horseradish peroxidase-conjugated secondary antibodies for 1h at room temperature. The results were recorded using an ImageQuant LAS 4000 mini imaging system (GE Healthcare, USA).

### Patients and Samples

Colorectal cancer samples were retrospectively collected with informed consent from patients undergoing surgery at PUMCH from November 2014 to December 2015. All the patients underwent R0 resection and were histologically diagnosed as having CRC. The last follow-up date for patients with no progression was December 2020. The study was approved by the Ethics Committee of the Chinese Academy of Medical Sciences and Peking Union Medical College. All patients signed written informed consent forms.

### Immunohistochemistry

Immunohistochemistry was performed using a standard protocol (29). The specimens were fixed with 10% formaldehyde, embedded in paraffin and sectioned into slides with 4μm thickness. The expression of PD-L1 (Abcam, UK), p-Akt (Cell Signaling Technology, USA), and α-SMA (Cell Signaling Technology, USA) were detected by immunohistochemistry (IHC). The slides were dewaxed and rehydrated, followed by antigen retrieval using microwaving in 0.01 mol/L citric acid buffer (pH 6.0). The endogenous peroxidase activity was blocked with 3% hydrogen peroxide at room temperature for 10 minutes. Goat serum was added as sealant and incubated for 20 minutes. The slides were incubated with primary antibodies at 4°C overnight. On the next day, the slides were incubated with the secondary antibody (Cell Signaling Technology, USA) at room temperature for 2 hours. Finally, the sections were observed under a microscope.

The results of IHC of all slides were reviewed independently by two pathologists who were blinded to the clinical data. If the results were inconsistent, a third pathologist was called to make the final decision. The percentage and intensity of PD-L1, p-AKT and α-SMA expression in tumor cells was scored. The membrane staining of tumor cells ≥ 1% was defined as PD-L1 positive (30). The expression of p-AKT and α-SMA were assessed using a previously published semiquantitative method (31).

### Statistical Analysis

The statistical analysis was conducted using SPSS 25.0 (IBM, Armonk, NY, USA) and GraphPad Prism 8(California, USA). The correlation between the expression of PD-L1, p-AKT, α-SMA and clinicopathological features was tested using the χ^2^ test. The relationship between the expression of PD-L1, p-AKT, α-SMA and disease-free survival (DFS) time was tested using the Kaplan-Meier method and the survival curve was plotted. DFS refers to the time from the R0 resection to disease recurrence demonstrated by imaging. Cytotoxicity to tumor cells was analyzed by two-way ANOVA. The two-sided probability test was adopted, with a significance level of P =0.05. Differences with P < 0.05 were considered statistically significant.

## Results

### CRC-Exosomes Induced the Differentiation of hAD-MSCs Into CAFs

**Figure 1:**
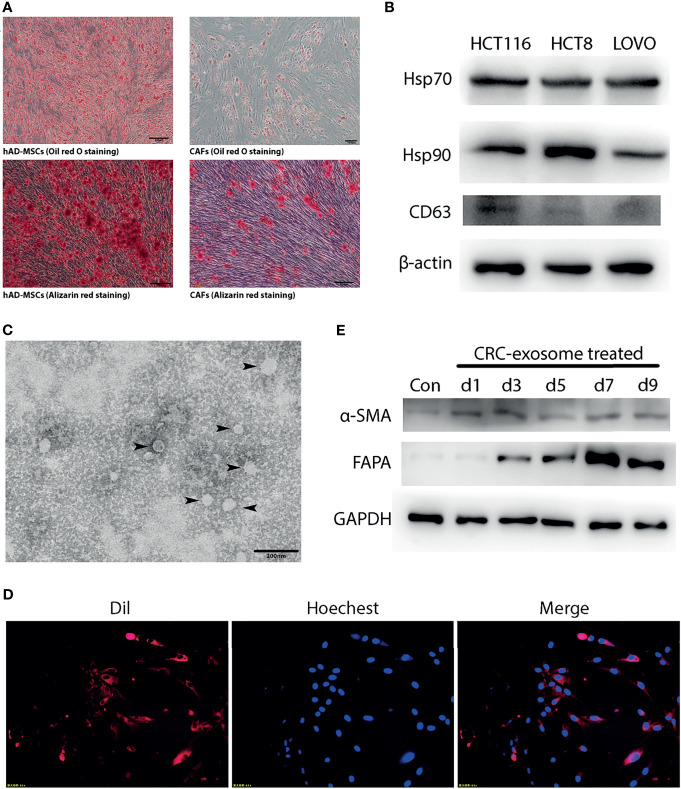
CRC-exosomes induced differentiation of hAD-MSCs into CAFs. **(A)** Comparison of adipogenic (Oil red O staining, day11) and osteogenic (Alizarin red staining, day14) differentiation ability between hAD-MSCs and CAFs. **(B)** Detection of CRC-exosomes specific marker proteins by Western Blot. **(C)** Ultrastructure of exosomes (black arrows) purified from HCT116 under TEM. **(D)** Exosomes from HCT116 (Dil-labeled) ingested by hAD-MSCs were verified with fluorescence microscopy after incubating for 4 hours. Red: Exosomes (Dil). Blue: Nucleus (Hoechst 33342). **(E)** Western Blot analysis showed that the expression of CAFs-specific proteins was upregulated after induction with CRC-exosomes.

To investigate the effect of CAFs on immune escape of CRC cell lines, we induced the differentiation of hAD-MSCs into CAFs *in vitro* using exosomes from CRC cell lines. We isolated hAD-MSCs from human adipose tissue according to our previous protocol (25). The hAD-MSCs expressed CD29, CD44, CD73, and CD90 (**Supplementary Figure 1A**), and had the ability of adipogenic and osteogenic differentiation (**Figure 1A**).

CRC-exosomes were purified from HCT8, HCT116 and LOVO cell lines, respectively. Western blot analysis showed that CRC-exosomes expressed Hsp70, Hsp90, and CD63 (**Figure 1B**). Exosomes purified from HCT116 cells presented as vesicles with a bilayer membrane structure with an average size of 40-100nm under TEM (**Figure 1C**). To examine whether CRC-exosomes can be taken up by hAD-MSCs, exosomes from HCT116 cells were stained with Dil and incubated with the hAD-MSCs. The results of fluorescence microscopy indicated that the exosomes were taken up into the cytoplasm (**Figure 1D**). After induction by CRC-exosomes, the expression of the marker proteins α-SMA and FAPα in hAD-MSCs was upregulated in a time-dependent manner (**Figure 1E**). The morphological changes of MSCs induced by CRC-exosomes over time were shown in **Supplementary Figure 1B**. As shown in **Figure 1A**, the pluripotency of hAD-MSCs was significantly decreased after they were induced to differentiate into CAFs. Therefore, it was demonstrated that CRC-exosomes can induce the differentiation of hAD-MSCs into CAFs.

### CAFs Promote the Immune Escape of CRC Cell Lines Through PD-L1 Upregulation

**Figure 2:**
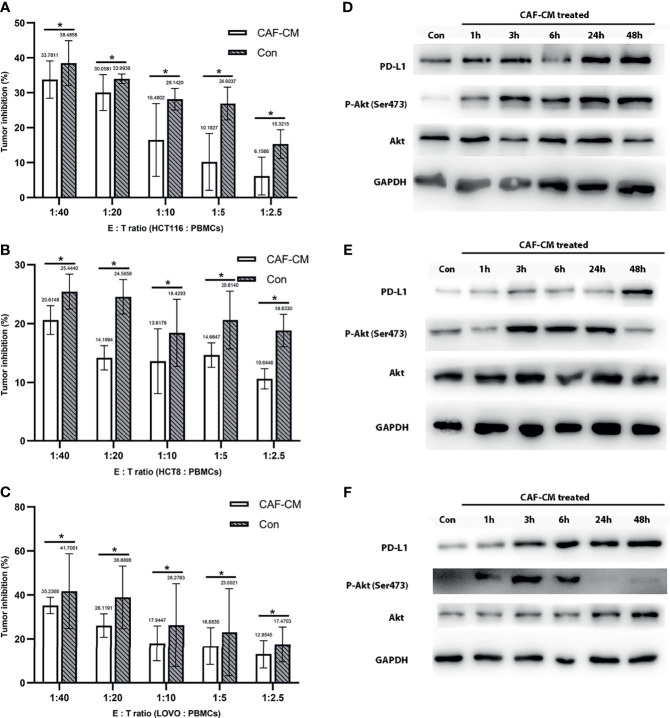
CAFs promote the immune escape of CRC cell lines through PD-L1 upregulation. **(A-C)** The killing rate of HCT116, HCT8, and LOVO cultured with CAF-CM for 72h was significantly lower than cells cultured with normal culture medium (Con). **(A)** HCT116; **(B)** HCT8; **(C)** LOVO. **(D–F)** The expression of PD-L1 and p-Akt on HCT116 **(D)**, HCT8 **(E)** and LOVO **(F)** was upregulated after treatment with CAF-CM. Each group was replicated in 6 wells. *P < 0.05.

To verify that CAFs promote the immune escape of tumor cells, we collected the conditioned medium of CAFs (CAF-CM) from HCT116 cells. We cultured HCT116 cells using CAF-CM and normal medium respectively, and then analyzed the killing rate of PBMCs with CAF-CM-treated HCT116 cells and control group after 3 days. Our data demonstrated that HCT116 cells treated with CAF-CM were significantly more resistant to killing by PBMCs compared with the control group (**Figure 2A**). We conducted the same experiment using HCT8 and LOVO cells, and the results were consistent (**Figures 2B, C**). To further confirm the result, we performed flow cytometry to detected the apoptosis ratio of CRC cells. We cultured HCT116 with CAF-CM or normal medium respectively for 48h. PBMCs were then added and co-cultured with tumor cells. The apoptosis kit was used for detection after 2 days. The results indicated that the apoptosis of tumor cells cultured using CAF-CM was less than that of control group (**Supplementary Figure 2**) which was consistent with the results of cytotoxicity assay. These results indicated that CAFs promoted the immune escape of CRC cell lines.

After finding that CAFs can promote the immune escape of CRC cells, we inferred that this may be mediated by the upregulation of the immune checkpoint protein PD-L1, similar to previous studies (16–18). First, we detected the effect of CAF-CM on the expression of PD-L1 in HCT8, HCT116 and LOVO cells, and found that the expression of PD-L1 was significantly upregulated 3 hours after CAF induction (**Figures 2D–F** and **Supplementary Figure 3**). While the expression of PD-1 on PBMCs did not change significantly after co-culture with CRC cell for 2 days (**Supplementary Figure 4**).

### CAFs Upregulated PD-L1 in CRC Cells Through Akt Phosphorylation

**Figure 3:**
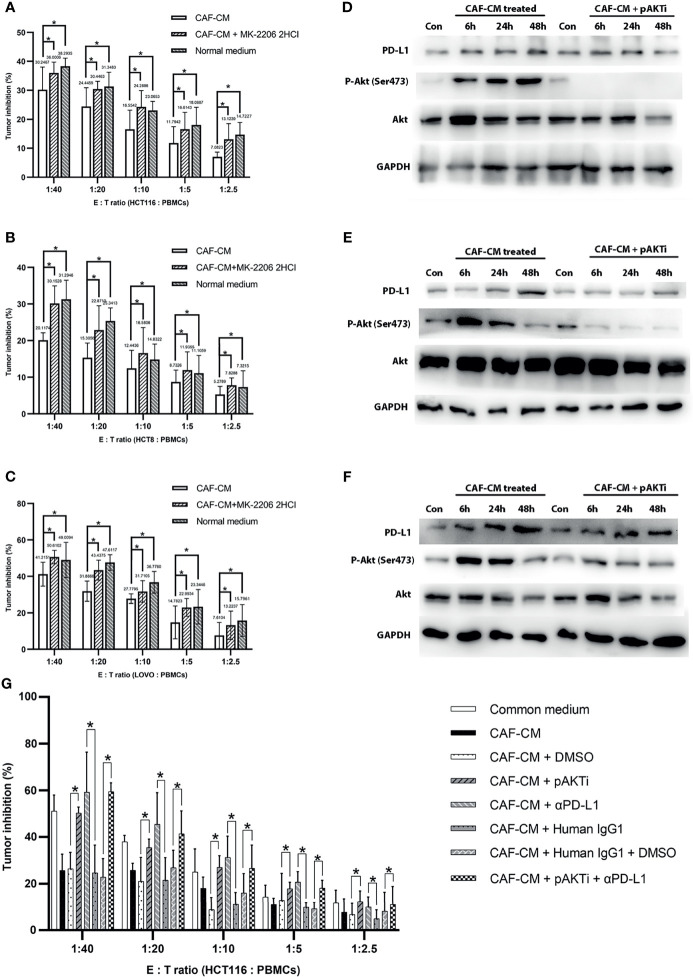
CAFs upregulated PD-L1 in CRC cells through Akt phosphorylation and promoted immune escape. **(A–C)** The killing rate of HCT116, HCT8 and LOVO cells cultured with CAF-CM was significant lower than these cells cultured with CAF-CM + pAKTi or normal medium after 72h. **(A)** HCT116; **(B)** HCT8; **(C)** LOVO. **(D–F)** The upregulation of PD-L1 expression on HCT116 **(D)**, HCT8 **(E)** and LOVO **(F)** was weakened compared with control groups after pAKTi (MK-2206 2HCI) was added. **(G)** After blocking of PD-L1, the killing rate was significant higher in CAF-CM + αPD-L1 group compared with CAF-CM + human IgG1 group. When both AKT and PD-L1 were blocked, the killing rate was similar to the CAF-CM + human IgG1 + DMSO group. There was no significant difference between the CAF-CM + pAKTi + αPD-L1 group with CAF-CM + pAKTi group or CAF-CM + αPD-L1 group. Each group was replicated in 6 wells. *P < 0.05. Con means cells treated with normal medium.

Since the upregulation of PD-L1 in CRC cell lines was fast, we inferred that the upregulation might be due to phosphorylation of proteins in signaling pathways. Previous studies have shown that CAFs promote the progression of various tumors by activating the Akt signaling pathway, so we detected the expression and phosphorylation of Akt in CRC cells treated with CAF-CM (32–35). We found that the phosphorylation of Akt in HCT8, HCT116 and LOVO cells was significantly increased at 1h, while the total Akt protein level was practically unchanged (**Figures 2D–F**). To prove the relationship between Akt phosphorylation and PD-L1 upregulation, we added the Akt phosphorylation inhibitor (pAKTi) MK-2206 2HCl into the medium. After inhibiting Akt phosphorylation, the upregulation of PD-L1 expression by CAF-CM was markedly weakened (**Figures 3D–F**). These results demonstrated that Akt phosphorylation is necessary for the CAF-induced upregulation of PD-L1 expression in CRC cell lines.

To further verify that CAFs promote the immune escape of colorectal cancer cells by upregulating the expression of PD-L1 through Akt phosphorylation, we cultured CRC cells with CAF-CM, CAF-CM + pAKTi, and normal medium, and the rate of killing by PBMCs was detected after 3 days. The results showed that the killing rate of CRC cells by PBMCs was significantly increased after inhibiting Akt phosphorylation in CRC cells compared with the CAF-CM group, which was similar to the CRC cells cultured in normal medium (**Figures 3A–C**). These results indicated that CAFs may promote the immune escape of CRC cells by upregulating Akt phosphorylation.

To investigate the exact contribution of PD-L1 to immune escape, we performed PD-L1 pre-blockade on HCT116 before cytotoxic assay using PD-L1 blocker(αPD-L1) atezolizumab biosimilar with/without pAKTi. We detected the blocking efficiency of αPD-L1 against PD-L1 by flow cytometry according to a published method (36), and then selected the concentration of αPD-L1 with the blocking efficiency of 90% for the following experiment. When PD-L1 was blocked, the killing rate was significant higher in CAF-CM + αPD-L1 group compared with CAF-CM + human IgG1 group (**Figure 3G**). Consistent with above results, tumor cells in CAF-CM + pAKTi group were significant more vulnerable to killing by PBMCs than CAF-CM + DMSO group (**Figure 3G**). When both AKT and PD-L1 were blocked, the killing rate was similar to the CAF-CM + human IgG1 + DMSO group (**Figure 3G**). There was no significant difference between the CAF-CM + αPD-L1 group with CAF-CM + pAKTi group, suggesting they could be in the same signaling pathway. In the meanwhile, the results of killing assay for CAF-CM + pAKTi + αPD-L1 group, CAF-CM + pAKTi group and CAF-CM + αPD-L1 group were similar. The results further manifested that the phosphorylation of AKT acted upstream of PD-L1, mainly affecting PD-L1 expression. When PD-L1 expression was neutralized, AKT phosphorylation blocking would not work.

### Clinical Characteristics of Patients

**Figure 4:**
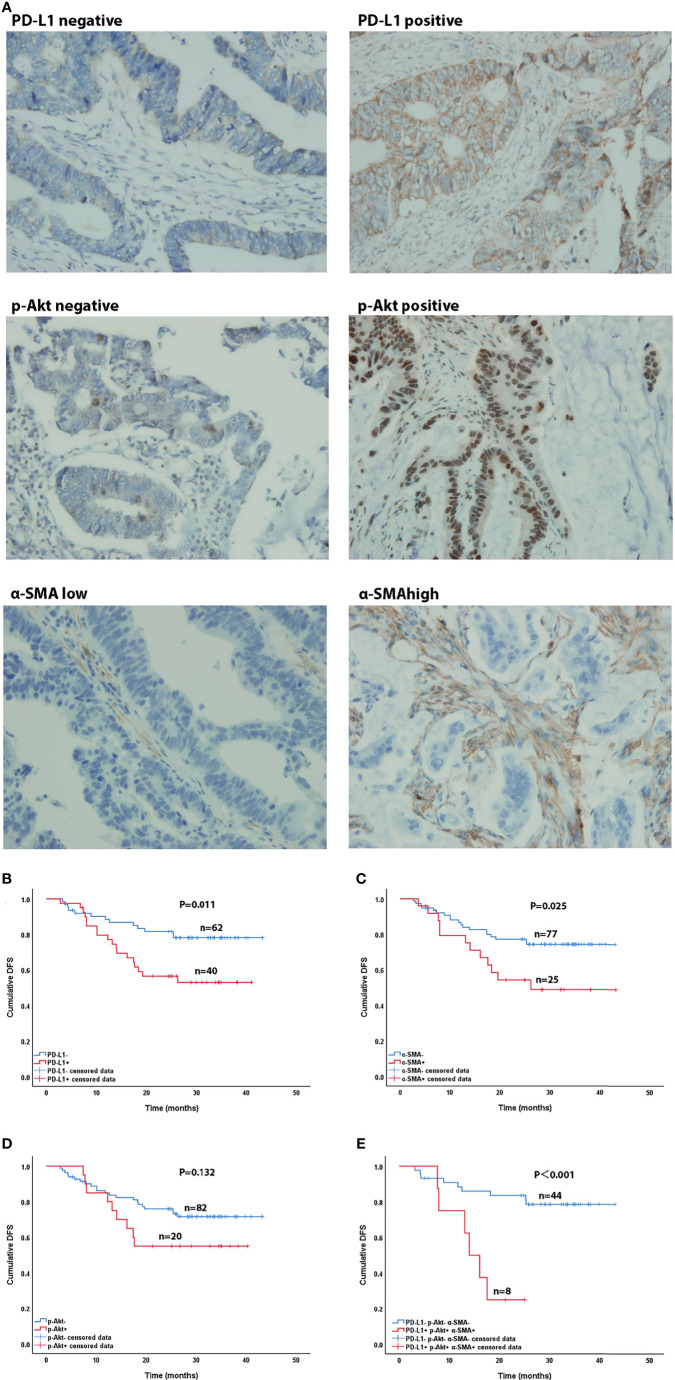
Immunohistochemical (×400) and Kaplan-Meier curves of CRC patients. **(A)** Immunohistochemical staining of PD-L1, p-AKT and α-SMA expression. **(B–D)** Effect of PD-L1 expression **(B)**, p-AKT expression **(C)**, and α-SMA high expression **(D)** on DFS in CRC patients. **(E)** Effect of co-expression of PD-L1, p-AKT and α-SMA high and triple-negative expression on DFS in CRC patients.

After *in vitro* experiments confirmed that CAFs can promote the phosphorylation of Akt in CRC cell lines, thereby upregulating the expression of PD-L1 and leading to immune escape of CRC, we investigated the correlations of PD-L1, CAFs, and p-Akt with the prognosis of CRC patients. A total of 102 postoperative patients with colorectal cancer were enrolled, including 49 males and 53 females. The clinical characteristics of the patients are summarized in **Table 1**. We determined the expression of PD-L1, p-Akt, and the commonly used marker protein α-SMA using IHC. Among 102 enrolled patients, 40 patients (39.2%) were positive for PD-L1 expression in the membrane of tumor cells, 20 (19.6%) were positive for p-AKT in the cytoplasm of tumor cells, and 25 (24.5%) had high α-SMA expression in the stromal cytoplasm (**Figure  4A**). Positive PD-L1 expression was associated with inferior tumor stage (χ^2 =^ 7.808, P=0.005). There was no significant correlation of PD-L1, p-AKT or α-SMA expression with age, gender, tumor location and tumor differentiation (P>0.05; **Table 1**). Correlation analysis suggested that the expression of PD-L1 was positively correlated with the p-AKT (Spearman R=0.213, P=0.031) and α-SMA levels (Spearman R=0.246, P=0.012). These data demonstrated that the expression of PD-L1 in CRC tissues was correlated with p-AKT and α-SMA.

**Table 1:** Relationship between PD-L1, p-AKT and α-SMA expression and clinicopathological characteristics in 102 patients with CRC.

	n	PD-L1 expression		χ2	P		p-AKT expression		χ2	P	α-SMA expression		χ2	P
		Positive (n)	Negative (n)				Positive (n)	Negative (n)			High (n)	Low (n)		
**Gender**				0.525		0.469			1.695	0.193			0.208	0.648
Male	49	21	28				7	42			13	36		
Female	53	19	34				13	40			12	41		
**Age (years)**				0.458		0.498			1.494	0.222			0.836	0.361
≤60	32	11	21				4	28			6	26		
>60	70	29	41				16	54			19	51		
**Location**				5.284		0.071			0.786	0.675			1.581	0.454
Right colonic carcinoma	77	26	51				14	63			17	60		
Left colonic carcinoma	24	14	10				6	18			8	16		
Right and left	1	0	1				0	1			0	1		
**Differentiation**				1.975		0.578			7.657	0.054			2.371	0.499
Highly differentiated	22	11	11				0	22			3	19		
Moderately differentiated	70	26	44				17	53			19	51		
Poorly differentiated	5	2	3				2	3			2	3		
Others	5	1	4				1	4			1	4		

### Correlation of PD-L1, p-AKT, and α-SMA Levels With Disease-Free Survival (DFS) of CRC Patients

After our data showed a correlation between PD-L1, CAFs, and p-Akt, we further explored their correlations with the DFS of CRC patients using Kaplan-Meier curve analysis. As shown in **Figures 4B–E**, the DFS of patients with positive PD-L1 expression [27.8 months, 95% confidence interval (CI)=23.3-32.4] was significantly shorter than that of patients with negative expression (36.5 months, 95%CI=33.2-39.9, P=0.011) (**Figure 4B**). Patients with high α-SMA expression (28.0months, 95%CI=21.7-34.3) had shorter DFS than those with low expression (35.2 months, 95%CI=32.1-38.4, P=0.025) (**Figure 4C**). The DFS of p-Akt positive patients tended to be worse than that of negative patients, but the difference was not significant (27.8 vs. 34.6 months, P=0.132) (**Figure 4D**). The DFS of patients with combined PD-L1^+^, p-AKT^+^ and α-SMA^high^ status (15.8 months, 95%CI=11.5-20.1) was remarkably shorter than that of patients with triple negative expression (36.7 months, 95%CI=32.8-40.6, P<0.001) (**Figure 4E**). These results showed that high expression of PD-L1 and α-SMA had a negative effect on the DFS of CRC patients.

## Discussion

As a vital element of the TME, CAFs play an essential role in cancer progression by releasing various immunosuppressors and regulating the composition of the extracellular matrix (19, 20). Notably, CAFs promote immune escape through various mechanisms, including enhancing the activity of myeloid-derived suppressor cells and regulatory T cells, upregulating the expression of Fas ligand (FasL) and PD-L1, as well as downregulating the expression of the major histocompatibility complex (MHC) (18). In this study, we found that CAF-CM could decrease the killing of cultured CRC cell lines by PBMCs, which suggested that CAFs may promote the immune escape of CRC.

CAFs were found to upregulate the expression of PD-L1 in cancer cells and promote tumor development in previous studies (17, 21, 23). The α-SMA protein is a commonly used marker of CAFs (24). Li et al. confirmed that the expression of PD-L1 in colorectal and melanoma tissues was positively correlated with the expression of α-SMA (17). It has also been reported that α-SMA^+^ CAFs from human colon cancer can express PD-L1 and significantly inhibit T cell proliferation (16). To validate the expression of PD-L1 and its role in CAFs, we detected the expression of PD-L1 and α-SMA in CRC cell lines and CRC samples. The IHC results showed that PD-L1 was expressed in CRC tumor cells. Among the enrolled patients, 39.2% had tumors with positive PD-L1 expression and 24.5% exhibited high α-SMA expression. The correlation analysis suggested that the expression of PD-L1 was correlated with the high expression of α-SMA. We confirmed that the expression of CAF and PD-L1 was positively correlated in CRC. Our cell culture experiments showed that CAFs can continuously upregulate PD-L1 expression in CRC cell lines, indicating that CAFs may promote the expression of PD-L1 in CRC.

In addition, many previous studies have shown that PD-L1 was upregulated and acted as an independent predictive factor of worse prognosis in a variety of tumors, including non-small cell lung cancer, breast cancer, melanoma and renal cancer (37–40). Our results showed that the expression of PD-L1 (P = 0.011) and high expression of α-SMA (P=0.025) were associated with a significant reduction in the DFS of the patients with resectable colorectal cancer, similar to the results reported previously (41–45). We inferred that downregulating the expression of PD-L1 may reduce immune escape and increase the killing of tumor cells by PBMCs. These findings indicate new potential targets for antitumor therapy related to the regulation of PD-L1 expression by the TME.

We further explored the mechanism by which CAFs promoted the upregulation of PD-L1 expression in CRC. According to the current literature, the expression of PD-L1 is regulated by multiple mechanisms, including (1) transcriptional regulation: MYC was reported to bind to the promoter region of PD-L1, increasing the expression of PD-L1 mRNA and protein (46, 47). It has been found that hypoxia−inducible factor−1 (HIF-1) could bind with hypoxic response element (HRE) and upregulate the PD-L1 expression, simultaneously cause T-cell apoptosis and function inhibition (48, 49). STAT3 and NF-κB were also important transcription factors upregulating PD-L1 (4). (2) Phosphorylation regulation of signal pathway: The hyperactive oncogenic pathways that regulate the expression of PD-L1 mainly include the phosphoinositide 3-kinase (PI3K)/Akt pathway, mitogen-activated protein kinase (MAPK) pathway, Janus protein tyrosine kinase/signal transducer and activator of transcription (JAK/STAT) pathway and NF-κB pathway (4, 50–52). (3) Epigenetic regulation: MicroRNAs (miRNAs), such as miR513 (53), miR570 (54), miR142 (55) and miR200 (56), were also pivotal regulators of PD-L1 at epigenetic level. As for CAFs, it has been shown to promote the expression of PD-L1 by secreting CXCL5 in mice cancer cells (57). Dou D. et al. found high level of miR92 in CAFs-derived exosomes and the expression of PD-L1 was regulated after treating with the exosomes (58). But the mechanisms were not entirely clear due to the complex composition of CAF-CM.

Previous studies have demonstrated that CAFs activate the Akt signaling pathway in tumor cells to promote the progression of various cancers, including CRC, gastric cancer and lung cancer (32–35). Moreover, the expression of PD-L1 was significantly upregulated in CRC cell lines within a short period of time under the action of CAFs in our study. Consequently, we analyzed the phosphorylation status of the Akt signaling pathway and found that the levels of Akt and p-Akt were increased after treatment of CRC cell lines with CAFs. Conversely, blocking Akt phosphorylation with the specific inhibitor MK-2206 2HCI markedly reduced PD-L1 expression and significantly improved the killing rate of CRC cells by PBMCs. These results indicated that, in addition to the reported mechanisms, CAFs may induce immune escape of CRC by upregulating PD-L1 expression through Akt phosphorylation.

Although this study confirmed the correlation of PD-L1 and p-AKT expression with clinical characteristics and patient prognosis, it still has limitations due to a lack of enrolled patients in stage I and stage IV. The conclusions of this study should be confirmed by enrolling more patients in the future.

## Conclusions

In conclusion, we found that CAFs may promote the expression of PD-L1 in tumor cells *via* the Akt signaling pathway, leading to immune escape in CRC. Furthermore, the expression of PD-L1 was correlated with higher TNM stage and shorter DFS in CRC patients. PD-L1 and p-AKT may be potential targets for combined therapy in CRC.

## Data Availability Statement

The original contributions presented in the study are included in the article/**Supplementary Material**. Further inquiries can be directed to the corresponding authors.

## Ethics Statement

The studies involving human participants were reviewed and approved by Ethics Committee of the Chinese Academy of Medical Sciences and Peking Union Medical College. The patients/participants provided their written informed consent to participate in this study.

## Author Contributions

YG, ZS, JG, ZL, XX, CX, and XL conducted the experiments, acquired the data, and wrote the manuscript. LZ and JZ contributed to the collection and analysis of clinical data. CB, QH, and RC designed the experiments and supervised the study. All authors contributed to the article and approved the submitted version.

## Conflict of Interest

The authors declare that the research was conducted in the absence of any commercial or financial relationships that could be construed as a potential conflict of interest.

## Publisher’s Note

All claims expressed in this article are solely those of the authors and do not necessarily represent those of their affiliated organizations, or those of the publisher, the editors and the reviewers. Any product that may be evaluated in this article, or claim that may be made by its manufacturer, is not guaranteed or endorsed by the publisher.

## Supplementary Material

Supplementary Figure 1 Phenotype and morphology of hAD-MSCs. **(A)** Phenotype of hAD-MSCs detected by flow cytometry. **(B)** Morphology changes of MSCs over time after treating with CRC-exosomes.

Supplementary Figure 2 Apoptosis of HCT116 cells co-cultured with PBMCs after treatment with CAF-CM or normal medium. **(A)** The apoptosis of HCT116 cultured using CAF-CM was significant less than that of control group. **(B, C)** The results of HCT116 apoptosis detected by flow cytometry. *P<0.05

Supplementary Figure 3 Western Blot analysis showed that the upregulation of PD-L1 was maintained until 72h after treatment with CAF-CM.

Supplementary Figure 4 The expression of PD-1 on PBMCs didn’t change significantly after co-culture with CRC cell.
