# Supplementary figures and images for: Cancer-Associated Fibroblasts Promote the Upregulation of PD-L1 Expression Through Akt Phosphorylation in Colorectal Cancer
Source: Front Oncol. 2021 Nov 19;11:748465. doi: 10.3389/fonc.2021.748465 (PMC8640083; doi:10.3389/fonc.2021.748465)

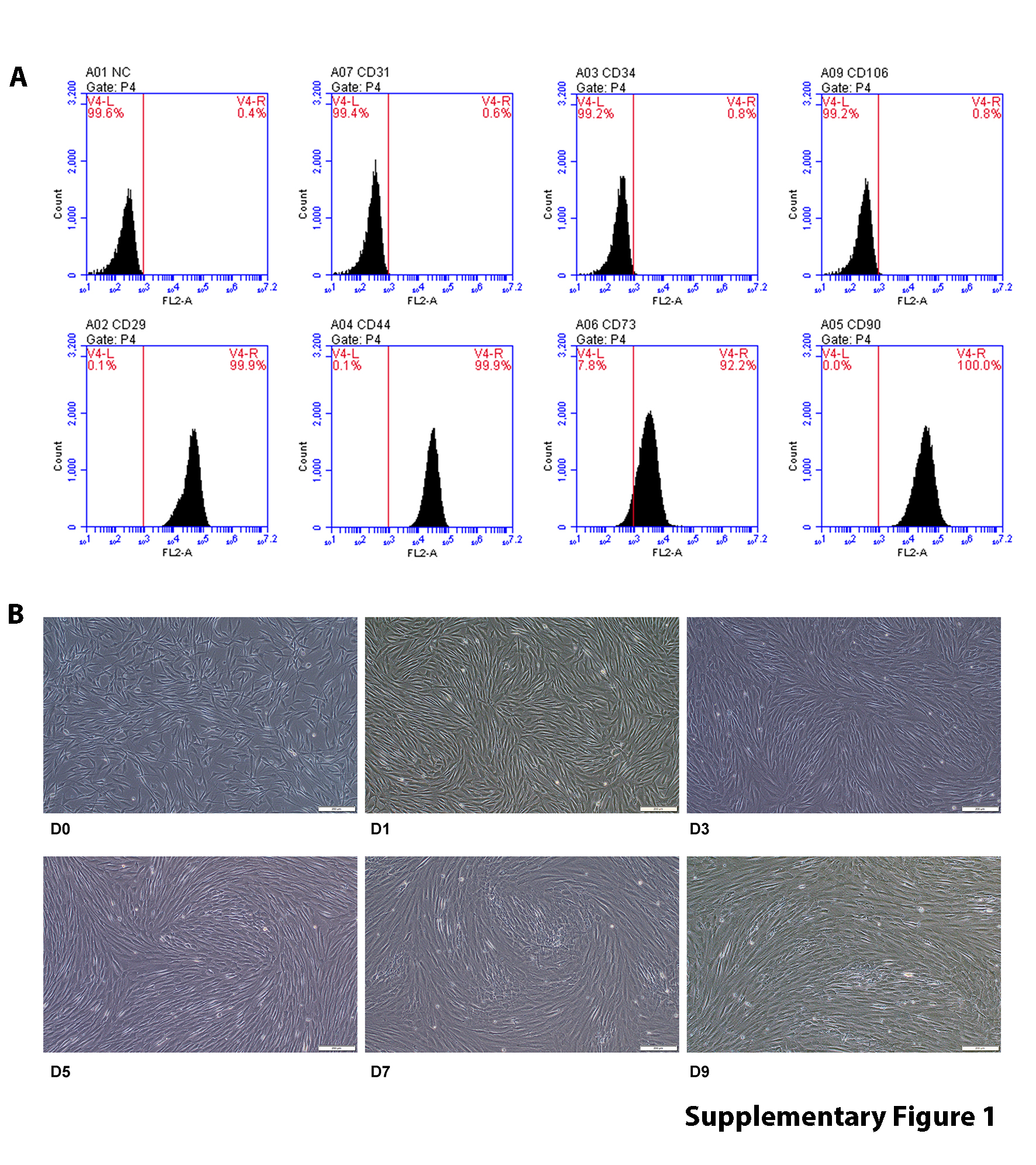

Supplement: Supplementary Figure 1 — Phenotype and morphology of hAD-MSCs. (A) Phenotype of hAD-MSCs detected by flow cytometry. (B) Morphology changes of MSCs over time after treating with CRC-exosomes. [file Image_1.jpeg]

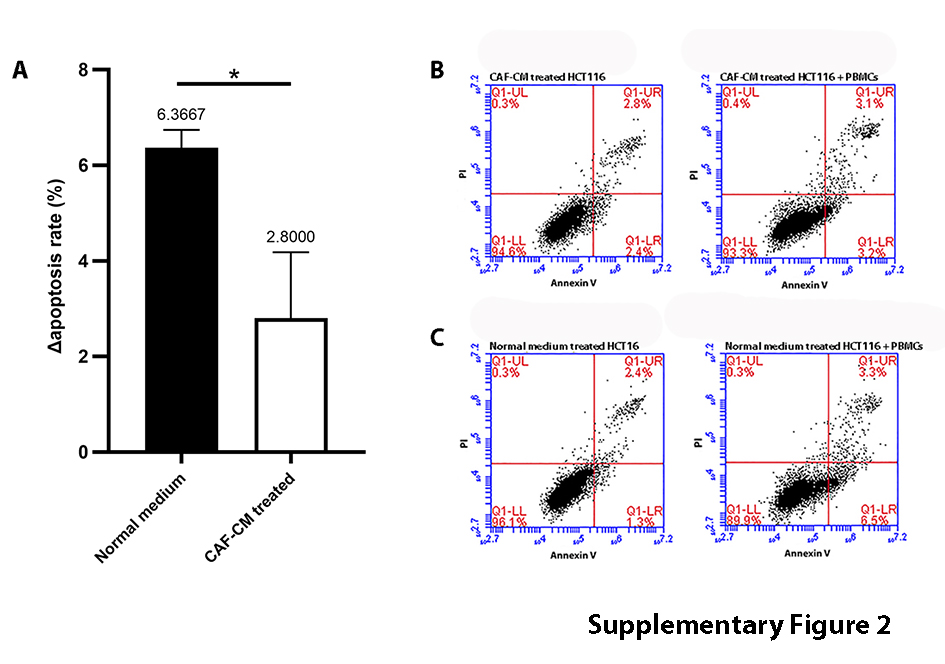

Supplement: Supplementary Figure 2 — Apoptosis of HCT116 cells co-cultured with PBMCs after treatment with CAF-CM or normal medium. (A) The apoptosis of HCT116 cultured using CAF-CM was significant less than that of control group. (B, C) The results of HCT116 apoptosis detected by flow cytometry. *P<0.05 [file Image_2.jpeg]

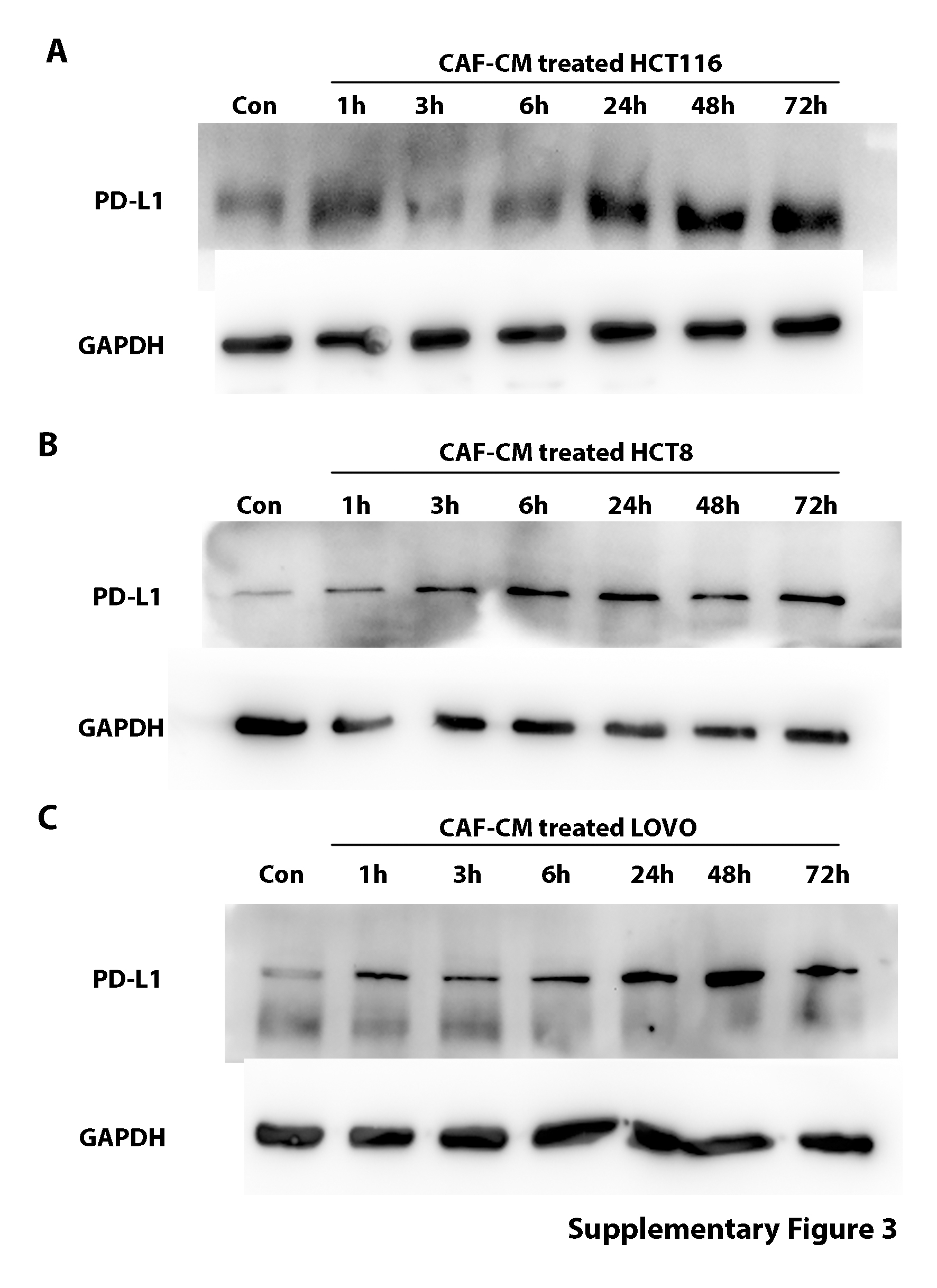

Supplement: Supplementary Figure 3 — Western Blot analysis showed that the upregulation of PD-L1 was maintained until 72h after treatment with CAF-CM. [file Image_3.jpeg]

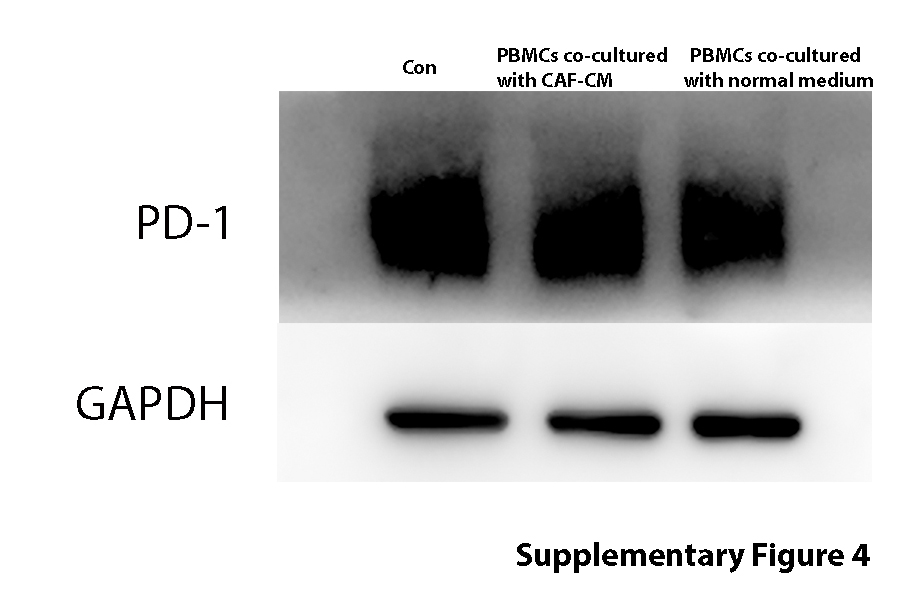

Supplement: Supplementary Figure 4 — The expression of PD-1 on PBMCs didn’t change significantly after co-culture with CRC cell. [file Image_4.jpeg]
